# Eco-friendly RP-HPLC method for simultaneous determination of water-soluble and fat-soluble vitamins in nano-formula and pharmaceutical dosage forms

**DOI:** 10.1186/s13065-025-01441-1

**Published:** 2025-03-20

**Authors:** Safaa Hussein Salah El-Din, Amr M. Mahmoud, Amany Morsi

**Affiliations:** 1 Egyptian Drug Authority, Cairo, Egypt; 2 Analytical Chemistry Department, Faculty of Pharmacy, Cairo University, Cairo, 11562 Egypt

**Keywords:** Vitamin A, Vitamin C, Water soluble vitamin, Fat soluble vitamin, RP-HPLC, Isocratic elution

## Abstract

A green method for simultaneous determination of water soluble vitamin (vitamin C) and fat soluble vitamin (vitamin A) was developed using reversed phase high performance liquid chromatography technique. The method succeed to separate the water-soluble and fat-soluble vitamins by isocratic elution using Agilent Zorbax octylsilane column (250 × 4.6 mm, 5 μm) in a short single run. The proposed mobile phase consisted of buffer (10 mM potassium dihydrogen phosphate and 3 mM hexane sulfonic acid sodium salt), pH adjusted to 2.5 using orthophosphoric acid and methanol in a ratio (8:92 v/v) with flow rate 1.0 mL.min^− 1^ and UV detection 328 nm for vitamin A and 243 nm for vitamin C in concentration range (0.5–30 IU.mL^− 1^) and (1–60 µg.mL^− 1^), respectively. Accuracy results were 99.49% ± 1.58 for vitamin C and 100.26% ± 1.86 for vitamin A, limit of detection (L.O.D) of vitamin C is 0.3 µg.mL^− 1^ while for vitamin A is 0.15 IU.mL^− 1^ and limit of quantification (L.O.Q) of vitamin C is 1.0 µg.mL^− 1^ while for vitamin A is 0.5 IU.mL^− 1^. Analytical eco scale and green analytical procedure index showed that our proposed method is greener than the reported method. The proposed method validation was performed according to ICH guidelines and the method was applied successfully for determination of vitamin A and vitamin C simultaneously in cosmetic nano-formulation, pharmaceutical dosage form and in pure forms.

## Introduction

The vitamins are low molecular weight organic substances which have key roles in metabolism. They are classified according to their solubility, either fat-soluble or water-soluble. The water-soluble vitamins have one or more ionizable polar groups (keto, carboxyl, hydroxyl, phosphate or amino), while the fat-soluble vitamins have aliphatic and aromatic characters. Fat-soluble vitamins are soluble in non-polar solvents: vitamins A, D, E and K. Water-soluble vitamins are soluble in polar solvents: vitamin B and vitamin C [1].

**Fig. 1:**
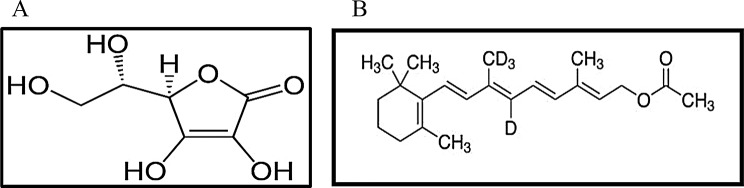
**a**: Ascorbic Acid Chemical Structure. **b**: Retinyl Acetate Chemical Structure

Vitamin C (Vit. C); L-Ascorbic acid is the enolic form of (2,3-didehydro-L-threo-hexano-1,4-lactone), Fig. 1a [2]. Vit. C is a powerful antioxidant compound that capture free radicals and it has potent antibacterial effects due to its low pH. Vit. C is able to inhibit the growth of *S. aureus* and streptococci even under neutral pH conditions [3]. Vitamin A Acetate (Vit. A); Retinol Acetate; Retinyl Acetate [2], All-trans-3, 7-dimethyl-9-(2, 6, 6-trimethyl-1-cyclohexen-1-yl)-2, 4, 6, 8- nonatetraene-1-yl acetate Fig. 1b [4]. Vit. A induces Resistin Like Molecule α (RELMa) which is an antimicrobial protein expressed by epidermal cells that limits skin infection and provides it with antimicrobial protection [5]. Vit. A has been used to treat various skin disorders like acne and psoriasis and in retinitis pigmentosa patients to enhance retinal function [2].

Literature review showed that vit. A and vit. C were determined simultaneously by High performance liquid chromatography (HPLC) method using isocratic and linear gradient elution in approximately 18 min [6]. Vit. C was determined by different techniques: HPLC [7–21], Voltammetry [22], Spectrophotometry [23] and Titration [24], While Vit. A was determined by HPLC [25–30] Capillary electrophoresis [31] and Spectrophotometry [32, 33]. The main aim of our work is to develop a simple chromatographic method for determination of both vit. A and vit. C accurately and with good resolution.That was done in short single run using isocratic elution in their pure form, nano-cosmetic formulation and in pharmaceutical dosage form without interference from the matrix (Vitamin E, Selenium, Maisine 35 − 1, Kolliphor RH-40 and Tween 80). This was a big challenge as vit. C is polar so it elutes very fast, while vit. A is non polar so it retains on the column. In previous work they depended on separating water-soluble vitamins and fat-soluble vitamins utilizing gradient elution which takes about 18 min [6] while our work separated both vitamins with isocratic elution in 6 min. The developed method was validated according to the ICH guidelines [34] showing that the results are accurate and precise. Greenness comparison between our method and the reported method [6] was done using different tools [35–42] and our developed method results showed that it is greener than the reported method.

## Experimental

### Instrumentation

Waters Arc HPLC consisted of quaternary pump, Diode array detector and automatic injector 50 µL loop (Waters, United States). The stationary phase was Agilent Zorbax octylsilane column (250 × 4.6 mm, 5 μm) (Agilent, United States).

### Materials and reagents

#### Chemical and reagents

Bi-distilled water; Milli-Q (Burlington, United States), orthophosphoric acid; El-Nasr Pharmaceutical Co. (Qalyubia, Egypt), methanol HPLC grade; Fisher Scientific (Loughborough, United Kingdom), potassium dihydrogen phosphate; TEDIA (Fairfield, United States), hexanesulfonic acid sodium salt; Loba Chemie (Mumbai, India).

#### Authentic samples

Ascorbic acid (99.90%) was purchased from Sigma aldrich (Burlington, United States), Retinyl acetate 500,000 IU.gm^− 1^ was supplied by Arab company for Pharmaceuticals and medicinal plants (Sharkeia, Egypt).

#### Pharmaceutical sample

Antox tab, Batch No.: 1,741,121, each tablet contains 2036.46 IU vit. A, 90 mg vit. C, manufactured by MEPACO– Egypt.

#### Nano-cosmetic formula

A formula was prepared in lab. as nano-emulsion, Batch No.: F7, each gm contains 9.8 mg vit. A and 9.8 mg vit. C.

### Standard solutions

Standard stock solution 100 IU.mL^− 1^ of vit. A and 200 µg.mL^− 1^ of vit. C were prepared separately in methanol (pH of methanol adjusted to 6.0 with orthophosphoric acid). We added small hot water drops to vit. A prior to adjusting to the mark with methanol for better dissolving of the powder.

### Laboratory prepared mixtures

Aliquot portions of standard stock solution of vit. A (100 IU.mL^-1^) and vit. C (200 µg.mL^-1^) were transferred into a series of 10-mL volumetric flasks to prepare mixtures with different ratios including the market ratio of the nano-formula (1: 1), the volume was completed with methanol (pH 6.0).

### Chromatographic conditions

Reverse-phase high-performance liquid chromatography (RP-HPLC) analysis was conducted using an Agilent Zorbax octylsilane column (250 × 4.6 mm, 5 μm) as the stationary phase. The separation was performed under isocratic elution conditions at room temperature. Employing a mobile phase composed of Solution A—a buffer solution containing 10 mM potassium dihydrogen phosphate and 3 mM hexane sulfonic acid salt, with pH adjusted to 2.5 using orthophosphoric acid—and Solution B, which consisted of methanol. The mobile phase was utilized in an 8:92 (v/v) ratio of Solution A to Solution B. The flow rate was maintained at 1 mL/min. The detection was carried out using UV absorbance at 243 nm for vit. C and 328 nm for vit. A. Sample injections were performed in triplicate, with an injection volume of 10 µL.

### Procedure

#### Construction of calibration curves

Aliquots of vit. C and vit. A from their standard stock solution (200 µg.mL^− 1^) and (100 IU.mL^− 1^), respectively, were transferred into two separate series of 10-mL volumetric flasks. The volumes were completed to mark with methanol (pH 6) to obtain concentration range of (1–60 µg.mL^− 1^) for vit. C at 243 nm, (0.5–30 IU.mL^− 1^) for vit. A at 328 nm. Then 10 µL of each solution was injected under operating conditions previously described in triplicates. Calibration curves representing the relationship between peak area and their corresponding concentrations in µg.mL^− 1^ for vit. C and IU.mL^− 1^ for vit. A were constructed and the regression equations were then computed.

#### Assay of cosmetic nano formulation

An accurate weight ~ 0.1 gm of cosmetic nano-emulsion prepared in the laboratory, Batch No.: F7, claimed to contain 9.8 mg/gm of vit. C and 9.8 mg/gm of vit. A (equivalent to 4900 IU in 1 gm) was weighed and transferred to a 50 mL volumetric flask. The volume was completed to the mark with methanol pH adjusted to 6.0 to get concentration of 19.6 µg.mL^− 1^ vit. C and 9.8 IU.mL^− 1^ vit. A, sonicated 5 min, and then filtered through 0.45 μm nylon syringe filter. The filtrate was analyzed in triplicates by the previous described method. The concentrations were calculated from the corresponding regression equations and the mean recovery % was then calculated.

#### Assay of pharmaceutical Preparation

An accurate weight ~ 28 mg of Antox tab., Batch No.: (1741121) claimed to contain 90 mg/tab vit. C and 2036.46 IU/tab vit. A was transferred to a 100 mL volumetric flask, small drops of hot water were added to the powder. Then the volume was completed to the mark with methanol pH adjusted to 6.0 to get concentration of 50 µg.mL^− 1^ vit. C and 1.13 IU.mL^− 1^ vit. A, sonicated 5 min, and then filtered through 0.45 μm nylon syringe filter. The filtrate was analyzed in triplicates by the previous described method. The concentrations were calculated from the corresponding regression equations and the mean recovery % was then calculated.

#### Greenness assessment of the developed method (Green metrics)

Green chemistry concept is very important now in chemical laboratories so assessment tools are required to measure the environmental impact of chemical procedures. In this paper we used three different green metrics to evaluate the greenness profile of the developed RP-HPLC method. The first tool is the “National Environmental Method Index” (NEMI) [35, 36], the second tool is the “Analytical Eco-Scale” method [37] and the third tool is “Green Analytical Procedure Index” (GAPI) tool [38].

#### National environmental method index (NEMI label)

It is a circle divided to four quadrants that help to assess the analytical procedure, when the quadrant is colored green so the requirements for this quadrant are met. The first quadrant requires that all chemicals used in the procedure are not listed on the (PBT) persistent, bioaccumulative and toxic chemicals list. The second quadrant requires that all chemicals used are not listed on D, F, P or U hazardous wastes lists. The third quadrant requires the pH of the sample is not corrosive within (2–12). The fourth quadrant requires that the produced waste is less than 50 g [35, 36].

#### Analytical Eco-Scale

Analytical Eco-Scale is a scale from 100 backward showing how green the method is. A score of 100 means an ideal green method. Penalty points, which represent aspects of the analytical procedure that deviate from the ideal green analysis, are subtracted based on factors such as the type and quantity of reagents used, associated hazards, energy consumption, and the volume of generated waste. An inadequate green method will score less than 50, acceptable green method scores more than 50 while an excellent green method scores more than 75 [37].

#### Green analytical procedure index (GAPI)

It is a pictogram that classify the greenness of each step of an analytical procedure by using a color scale green, yellow and red which represents low, medium and high environmental impact. In GAPI symbol five pentagrams are used to evaluate and quantify the analytical procedure. Each pictogram consists of different parts, each part represents a different step of the analytical procedure and the part is filled green when certain requirements are met [38–42].

## Results and discussion

### Method development

**Fig. 2:**
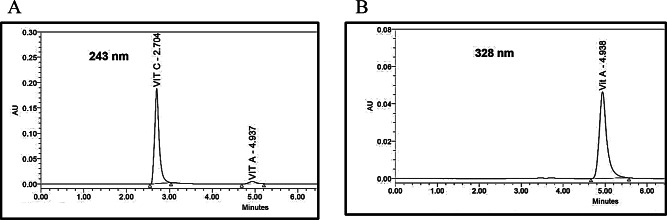
**a**: A typical HPLC chromatogram of a mixture of Vit. C and Vit. A, detection at 243 nm. **b**: A typical HPLC chromatogram of a mixture of Vit. C and Vit. A, detection at 328 nm

The combination of vit. C and vit. A has excellent role in skin care products especially in treating wrinkles and protecting skin against UV irradiation specially UVB cell damage and hyperpigmentation [43]. Nano formulation cosmetic products are mainly chosen because they overcome the common limitations of cosmetics. They enhance penetration and material dispersibility, stabilize ingredients, control active ingredients release, improve the products’ textural quality and functioning themselves as active agents because of their tiny dimensions together with the large surface area [44]. The previous reported quantification methods preferred to make a method for water-soluble vitamins and a method for fat-soluble vitamins, while the reported method which made simultaneous determination for both vitamins used gradient elution and this caused long run [6]. In our experiment we developed a separation method for both vitamins simultaneously using isocratic elution in short run time about 6 min. We faced many challenges as the fast elution of vit. C so we used hexanesulfonic acid which enhance retention of vit. C and symmetry of its peak and this was not achieved by using water. While choosing the suitable pH for elution we found that the acidic pH (2.5) of the mobile phase give the best retention and symmetry of vitamins peak also it prevents degradation of vit. C [8]. Vit. C is stable near pH 3.0 and pH 6.0 [45] while vit A. in pH (from 5.6 to 7.0) did not influence the vitamin ester’s stability and pH levels of 4.0 and 8.0 showed a decrease in the stability of vit. A ester [46]. Thus, we used Methanol pH 6.0 adjusted by orthophosphoric acid as diluent as by trials the acidic medium showed degradation of vit. A and basic medium showed degradation of vit. C. To choose the suitable column we tried cyano column but vit. C eluted rapidly with bad symmetry of both vitamins. Then, we tried octadecylsilane column (250 × 4.6 mm, 5 μm) vit. A retained in column for long time so the best separation was achieved on octylsilane column (250 × 4.6 mm, 5 μm) where both vitamins eluted in short run with optimum resolution. Different mobile phase compositions were tried and the optimal ratio was (92:8 v/v) methanol: buffer with flow rate 1 mL.min^− 1^ and UV detection at 243 nm for vit. C and 328 nm for vit. A. The average retention times (min) were found to be 2.7 ± 0.1 for vit. C and 4.9 ± 0.1 for vit. A, Fig. 2a and b.

### Method validation

The method was validated according to ICH guidelines including linearity, specificity, accuracy, precision, robustness, limit of detection (LOD), limit of quantification (LOQ) and system suitability [34].

#### Linearity

Good linearity was obtained in concentration range of (1–60 µg.mL^− 1^) of vit C. at 243 nm and (0.5–30 IU.mL^− 1^) of vit A. at 328 nm, Table 1.

#### Precision

The proposed method precision was checked by the analysis of six different cosmetic nano emulsion samples (at 100% of the assay analyte concentration 3 replicates each) 19.6 µg.mL^− 1^ vit. C and 9.8 IU.mL^− 1^ vit. A on the same day (Repeatability) and on three successive days (Reproducibility). The percentage of relative standard deviation was then calculated and the results obtained are listed in Table 1 and revealed high precision of the developed method.

#### Accuracy

Accuracy of the developed methods was ascertained by recovery of known, added amounts of analyte (placebo spiked with stock standard solutions, placebo composed of maisine 35 − 1, kolliphor RH-40, tween 80 and water) covering the specified range for the procedure (5–55 µg.mL^− 1^) for vit. C and (2.5–27.5 IU.mL^− 1^) for vit. A. The concentrations were calculated using the corresponding regression equations and then the mean recovery % was calculated. The results revealed high accuracy of the developed method, Table 1.

#### Limit of detection (LOD) and limit of quantification (LOQ)

LOD and LOQ were obtained by using signal to noise ratio approach, Table 1.

#### Specificity

It is the ability to quantify accurately the vitamin in the presence of components which may be expected to be present as matrix. It was evaluated by analyzing mixtures containing different ratios of vit. C and vit. A. The results ascertained the proposed methods specificity, Table 2.

#### Application to commercial cosmetic Preparation and pharmaceutical product

The suggested methods were applied to determine vit. C and vit. A in Nano-cosmetic formula and Antox tab., Batch No.: (225144). The results showed good recovery consistent with the labeled amounts, Table 2.

#### Standard addition technique

Known concentrations of vit. C standard (5–55 µg.mL^− 1^) and vit. A standard (2.5–27.5 IU.mL^− 1^) were added to placebo and analyzed in triplicates by the previous method, Table 2.

#### Robustness

Robustness of the developed methods was ascertained by studying the effect of small but deliberate change in experimental variables. Minor variations in the method variables were applied to check the best conditions for separation such as flow rate ± 0.1, UV detection wavelength ± 2.0 and mobile phase composition ± 1.0% and no significant change in results were detected upon application on method.

#### Statistical comparison with reported methods

Statistical analysis of the suggested method results and the reported method results [6] in Table 3 indicated no significant differences between the developed method and the reported methods as the calculated t- and F-values were less than the theoretical ones.

#### System suitability determination

The parameters of system suitability testing were calculated according to USP [47] to ensure the correct work of HPLC systems during the analysis. Precision (RSD%), resolution (R), tailing factor (T), capacity factor (K) and theoretical plates (N). Table 4.

### Greenness assessment of the developed method (Green metrics)

#### National environmental method index (NEMI label)

**Fig. 3:**
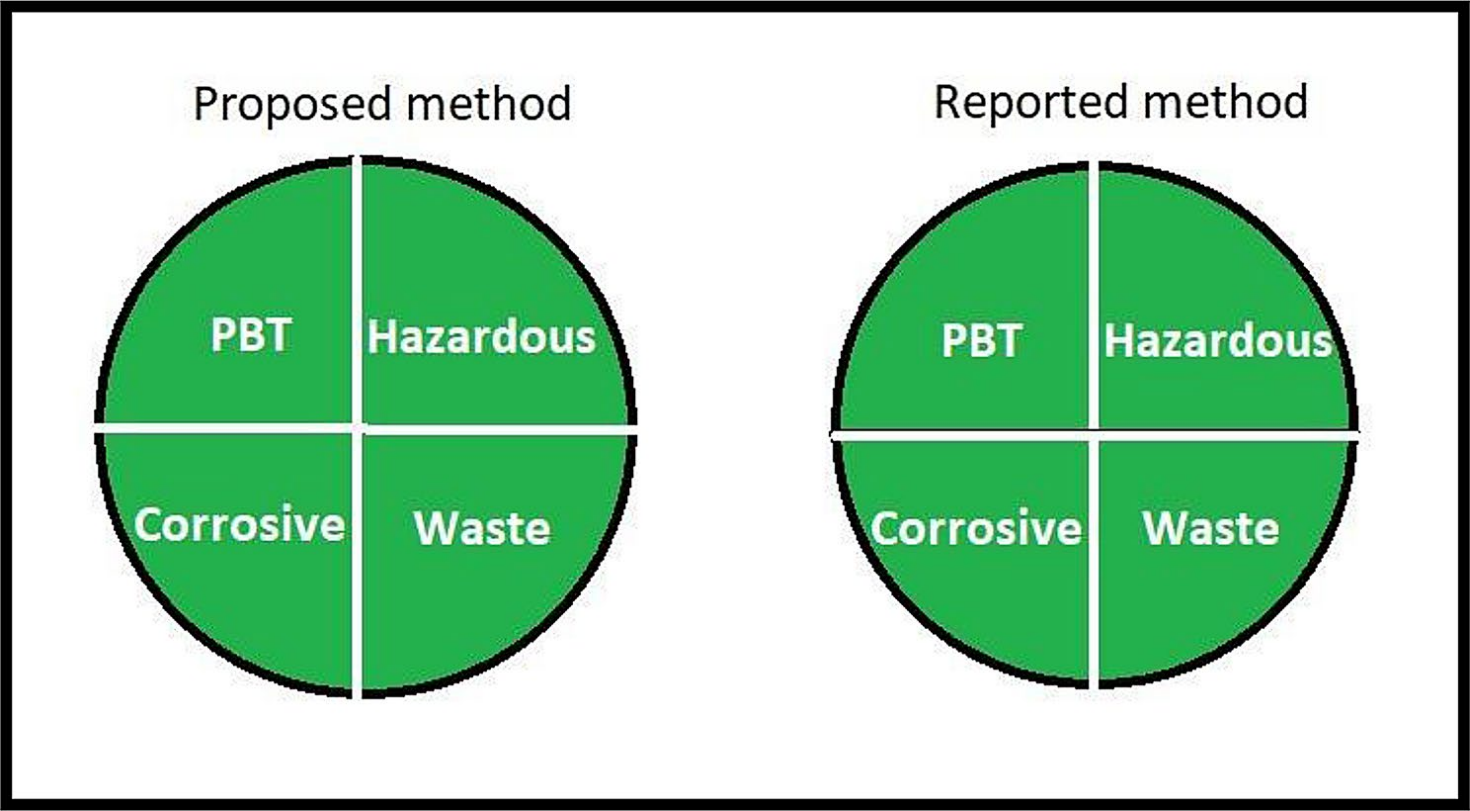
NEMI pictogram for the proposed HPLC method and reported HPLC method

The proposed method and reported method are green as the four quadrants of the circle met the required requirements as all chemicals used in both methods are not listed on (PBT) chemicals list [48] nor listed on D, F, P or U hazardous wastes lists [49], pH of the sample is 6 in the proposed method and 3.9 in the reported method and the produced waste in both methods is less than 50 g Fig. 3.

#### Analytical Eco-Scale

Analytical Eco-Scale score for the proposed RP-HPLC method and reported HPLC method is higher than 75 so both methods are excellent green methods but according to the analytical eco-scale score our proposed method is greener than the reported one. Tables 5 and 6.

#### Green analytical procedure index (GAPI)

**Fig. 4:**
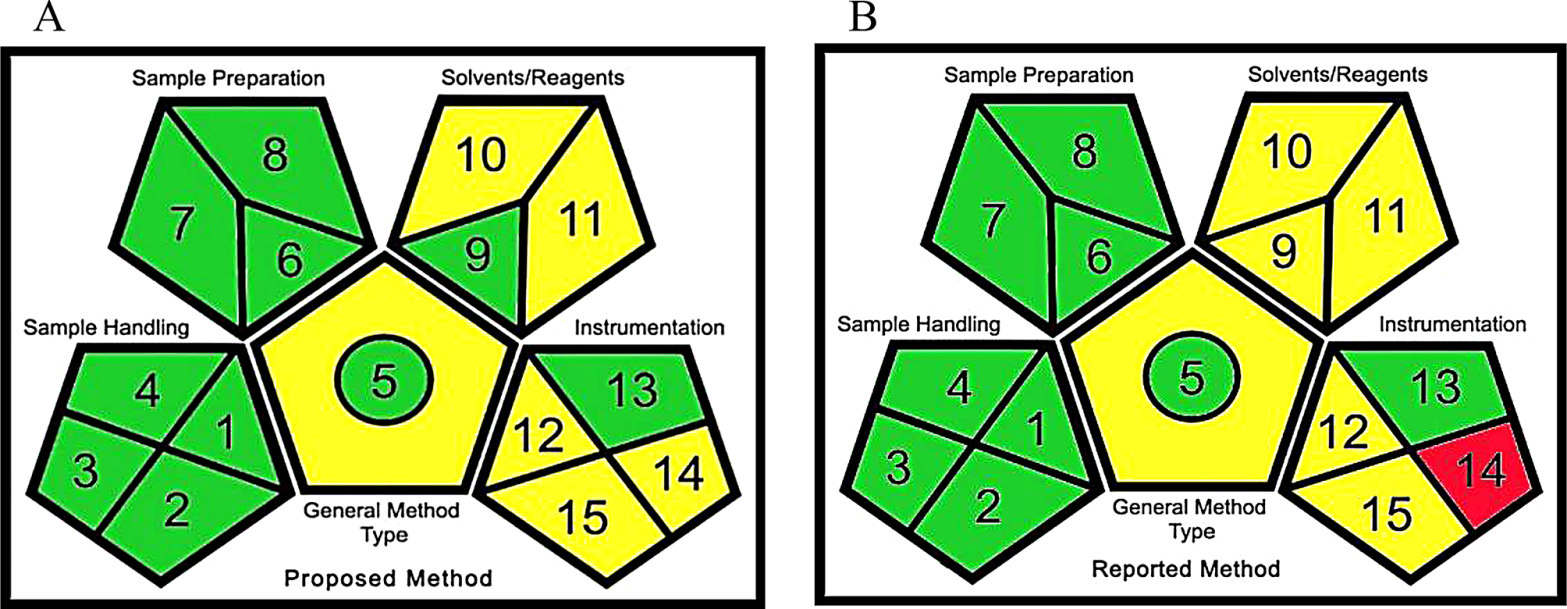
**a**:GAPI pictogram for the proposed method. **b**: GAPI pictogram for the reported method

GAPI pictograms in Fig. 4a and b showed that the proposed RP-HPLC method is greener than the reported HPLC method. Results are listed in Table 7.

**Table 1:** Assay validation parameters and regression equations for determination of vit. C and vit. A by the proposed chromatographic method

Parameter	Vit. C	Vit. A
Linearity	1–60 µg.mL^− 1^	0.5–30 IU.mL^− 1^
Regression equation	y = 34486x– 26,475	y = 23988x + 2052.4
Correlation coefficient (r)	0.9997	0.9999
Accuracy (Mean ± RSD)	99.49% ± 1.58	100.26% ± 1.86
LOD	0.3 µg.mL^− 1^	0.15 IU.mL^− 1^
LOQ	1 µg.mL^− 1^	0.5 IU.mL^− 1^
Repeatability ^(1)^	101.26% ± 0.91	98.27% ± 0.63
Reproducibility ^(2)^	101.41% ± 0.69	98.66% ± 0.89

(1) The intra-day (*n* = 18), Average of six different samples at 100% analyte concentrations repeated three times within day;

(2) The inter-day (*n* = 18), Average of six different samples at 100% analyte concentrations in three successive days

**Table 2:** Vit. C and vit. A determination in laboratory prepared mixtures, cosmetic nano-formulation, pharmaceutical Preparation and standard addition technique by the proposed methods

Drug Parameter	Vit. C	Vit. A
Laboratory prepared mixture ** (Mean ± RSD ) *n* = 6 *	99.78% ± 1.55	100.46% ± 1.11
Application in cosmetic formulation*** (Mean ± RSD ) *n* = 6*	100.59% ± 0.90	99.86% ± 1.27
Application in pharmaceutical preparation**** (Mean ± RSD ) *n* = 6*	100.72% ± 1.39	100.79% ± 1.21
Standard addition technique (Mean ± RSD ) *n* = 6*	99.49% ± 1.58	100.26% ± 1.86

* Average of triplicates measurements

** Average of one mixture repeated six times

*** Nano lotion formulation Batch No.: F7

**** Antox tab. Batch No.: 1,741,121

**Table 3:** Statistical analysis of the proposed method and the reported method [6] of vit. C and vit. A in the pure powder form

Drug	Method	Mean ± RSD	STDEV	VARIANCE	*N*	t-test (2.262) ^a^	F-ratio (6.26) ^a^
Vit. C	HPLC	99.49% ± 1.58	1.57	2.46	6	0.99	1.46
	Reported*	100.31% ± 1.30	1.30	1.69	6		
Vit. A	HPLC	100.26% ± 1.68	1.68	2.83	6	0.04	1.99
	Reported*	100.30% ± 1.19	1.19	1.42	6		

(a) Theoretical value for t-test and F-ratio for *P* = 0.05

* HPLC method using octadecylsilane column, mobile phase consisting of 0.010% trifluoroacetic acid of pH 3.9 and methanol in gradient elution at the flow rate 0.7 ml min^− 1^. And detection at 280 nm

**Table 4:** System suitability test results of the developed RP-HPLC method for determination of vit. C and vit. A simultaneously

Parameter	Vit. C	Vit. A
(RSD%) < 2	0.93	0.61
Resolution Rs > 2	-	8.69
Tailing factor T ≤ 2	1.30	1.14
Capacity factor K > 2	2.76	5.86
Plates Number *N* > 2000	4949	7730

**Table 5:** Analytical Eco-Scale score for proposed RP-HPLC method for vit. C and vit. A

Method item	Value			Penalty points
Methanol	Amount	5.52 mL	< 10 mL	Amount PP × Hazard PP 1*2 = 2
	Hazard	More sever hazard		
Hexane sulfonic acid	Amount	0.48 mL	< 10 mL	Amount PP × Hazard PP 1*0 = 0
	Hazard	none		
Orthophosphoric acid	Amount	< 1 mL	< 10 mL	Amount PP × Hazard PP 1*2 = 2
	Hazard	More sever hazard		
PH	2.5			0
Washing and conditioning time	normal			0
Waste	1–10 mL			3
HPLC	≤ 1.5 kWh per sample			1
Occupation Hazards	Emission of vapor to air			0
Total penalty points				8
Analytical eco scale score	≥ 75 excellent green method ≥ 50 acceptable green method < 50 inadequate green method			92

**Table 6:** Analytical Eco-Scale score for reported RP-HPLC [6] method for vit. C and vit. A

Method item	value				Penalty points
Methanol	Amount	15 mL		10–100 mL	Amount PP × Hazard PP 2 × 2 = 4
	Hazard	More sever hazard			
Trifluoroacetic acid	Amount	0.0006 mL	< 10 mL		Amount PP × Hazard PP 1 × 2 = 2
	Hazard	More sever hazard			
pH	3.9				0
Washing and conditioning time	normal				0
Waste	> 10 mL				5
HPLC	≤ 1.5 kWh per sample				1
Occupation Hazards	Emission of vapor to air				0
Total penalty points					12
Analytical eco scale score	≥ 75 excellent green method ≥ 50 acceptable green method < 50 inadequate green method				88

**Table 7:** Green analytical procedure index parameters description for proposed and reported [6] methods for determination of vit. C and vit. A

Category	Proposed Method			Reported Method	
	Type	Color		Type	Color
**Sample preparation**					
Collection(1)	In line	Green		In line	Green
Preservation (2)	None	Green		None	Green
Transport (3)	None	Green		None	Green
Storage (4)	None	Green		None	Green
Type of method: direct or indirect (5)	Simple procedure (filtration)	Yellow		Simple procedure (filtration)	Yellow
Scale of extraction (6)	None	Green		None	Green
Solvent/reagent used (7)	Green solvent	Green		Green solvent	Green
Additional treatment (8)	None	Green		None	Green
**Reagent and solvents**					
Amount (9)	< 10 ml	Green		10–100 ml	Yellow
Health hazard (10)	Moderate toxicity	Yellow		Moderate toxicity	Yellow
Safety hazard (11)	Flammable	Yellow		Flammable	Yellow
**Instrument**					
Energy (12)	≤ 1.5 kWh per sample	Yellow		≤ 1.5 kWh per sample	Yellow
Occupational hazard (13)	None	Green		None	Green
Waste (14)	< 10 mL	Yellow		> 10	Red
Waste treatment (15)	Passivation	Yellow		Passivation	Yellow
**Additional Mark: Quantification**					
Circle in the middle of GAPI	Yes Qualitative and quantitative method.		Yes Qualitative and quantitative method.		

## Conclusion

The proposed RP-HPLC method successfully determined both water-soluble vit. C and fat-soluble vit. A simultaneously, offering significant advantages over previously reported RP-HPLC methods. Unlike most literature methods, which employ separate procedures for each vitamin type, our method enables the simultaneous determination in a single run. A key improvement of our approach is the use of isocratic elution, whereas reported methods predominantly rely on gradient elution. This provides several benefits, including greater simplicity, more consistent retention times, elimination of the need for re-equilibration between runs, and a shorter run time of approximately six minutes without any interference, while maintaining high sensitivity. A statistical comparison between the results obtained using the developed method and those from reported methods demonstrated no significant difference, confirming the reliability of our approach. Additionally, the proposed method is greener, as evidenced by a higher analytical eco-scale score compared to the reported method. According to the GAPI pictogram, our method contains more green zones and no red zones, further confirming its environmental superiority. Given these advantages, the proposed method is suitable for application in quality control (QC) laboratories for the analysis of vit. C and vit. A in pure form, cosmetic formulations, and pharmaceutical preparations.

## Data Availability

The authors declare that the data supporting the findings of this study are available within the paper. Should any raw data files be needed in another format they are available from the corresponding author upon reasonable request.

## Abbreviations

Vit. A: Vitamin A
Vit. C: Vitamin C
ICH: International Council for Harmonization
RP-HPLC: Reversed Phase High Performance Liquid Chromatography
NEMI: National Environmental Method Index
GAPI: Green Analytical Procedure Index
LOD: Limit of Detection
LOQ: Limit of Quantification
UV: Ultraviolet
USP: United States Pharmacopeia
QC Lab: Quality Control Laboratory
